# Determinants of knowledge and attitudes toward personal protective equipment use among commercial motorcycle riders in a conflict-affected region of Cameroon: implications for road safety in Sub-Saharan Africa

**DOI:** 10.3389/fpubh.2026.1723195

**Published:** 2026-03-18

**Authors:** Chrisantus Eweh Ukah, Nicholas Tendongfor, Alan Hubbard, Elvis A. Tanue, Rasheedat Oke, Nahyeni Bassah, Larissa Kumenyuy Yunika, Sandra I. McCoy, Claudia Ngeha Ngu, Rebecca Hemono, S. Ariane Christie, Dickson Shey Nsagha, Alain Chichom-Mefire, Catherine Juillard

**Affiliations:** 1 Department of Public Health and Hygiene, Faculty of Health Sciences, University of Buea, Buea, Cameroon; 2 Division of Epidemiology, School of Public Health, University of California, Berkeley, Berkeley, CA, United States; 3 Program for the Advancement of Surgical Equity, Department of Surgery, University of California, Los Angeles, Los Angeles, CA, United States; 4 Department of Surgery and Specialties, Faculty of Health Sciences, University of Buea, Buea, Cameroon

**Keywords:** attitudes, Cameroon, displacement, knowledge, motorcycle safety, personal protective equipment, road traffic injury, Sub-Saharan Africa

## Abstract

**Background:**

Road traffic injuries are a leading cause of death globally, disproportionately affecting motorcyclists in low- and middle-income countries (LMICs). Personal protective equipment (PPE) such as helmets and visibility materials substantially reduce injury severity, yet their consistent use remains suboptimal in many African settings. In conflict-affected areas, where displacement and informal transport systems are common, safety behaviors may be further compromised. This study examined the determinants of knowledge and attitudes toward PPE use among commercial motorcycle riders in the Limbe and Tiko Health Districts of Cameroon to inform regional road safety interventions.

**Methods:**

A community-based cross-sectional study was conducted among 499 commercial motorcycle riders aged ≥18 years between May and August 2024. Participants were selected using a multistage sampling approach, and data were collected by trained research assistants through interviewer-administered structured questionnaires at randomly selected motorcycle pick-up points. The questionnaire captured sociodemographic characteristics, riding experience, and PPE knowledge (13 items) and attitudes (9 Likert-scale items). Knowledge and attitude scores were categorized using a 60% threshold. Multivariable logistic regression was applied to identify factors independently associated with good knowledge and positive attitudes.

**Results:**

The mean age of participants was 32.2 ± 7.6 years; all were male. Overall, 66.1% had good knowledge and 45.9% had positive attitudes toward PPE use. License ownership (AOR = 1.7; 95% CI: 1.1–2.6) and prior PPE training (AOR = 2.0; 95% CI: 1.4–3.0) were positively associated with knowledge, while internal displacement reduced the odds (AOR = 0.6; 95% CI: 0.4–0.9). Predictors of positive attitudes included license ownership (AOR = 1.8; 95% CI: 1.1–2.8), PPE training (AOR = 1.7; 95% CI: 1.1–2.6), and good knowledge (AOR = 10.4; 95% CI: 6.3–17.3). Internal displacement again reduced the likelihood of positive attitudes (AOR = 0.6; 95% CI: 0.4–0.9).

**Conclusion:**

Although knowledge of PPE was relatively high, attitudes remained inadequate, particularly among internally displaced riders. Strengthening motorcycle licensing systems, integrating PPE training into road safety programs, and addressing displacement-related vulnerabilities could improve safety behaviors. Findings from this conflict-affected region of Cameroon provide valuable insights for designing community-based interventions to enhance PPE use and reduce motorcycle-related injuries across Sub-Saharan Africa.

## Highlights

- Two-thirds of motorcycle riders in conflict-affected Cameroon had good PPE knowledge, but fewer than half had positive attitudes.
- License ownership and prior PPE training significantly improved both knowledge and attitudes.
- Internal displacement reduced the likelihood of safety awareness and compliance.
- Findings inform tailored interventions to improve PPE uptake in fragile and low-resource settings across Sub-Saharan Africa.

## Background

Road traffic injury is the eighth leading cause of death globally and is expected to be the 7th leading cause of death by 2030 (1). It accounts for approximately 1.3 million deaths per year, 92% of which occur in low- and middle-income countries, including Cameroon (2). In many low- and middle-income countries (LMICs), commercial motorcycle transport has become a vital means of livelihood and urban mobility, particularly in areas with inadequate public transportation systems (3). In low- and middle-income countries (LMICs), a significant proportion of road fatalities involve motorcyclists. Specifically, they account for approximately 39% of low-income countries and 34% of middle-income countries (4). In some regions, such as Southeast Asia, motorcycles are the greatest contributors to road traffic crashes, accounting for approximately 34% of deaths. Additionally, a recent study reported a notable increase in the proportion of fatalities involving motorcyclists in LMICs over the past decade, with a 44% increase reported (4). In Cameroon, motorcycles are a popular mode of transport in both rural and urban settings, offering flexible and affordable services (5). However, the rapid expansion of commercial motorcycle operations has been accompanied by an increase in road traffic injuries (RTIs), with motorcycle riders and their passengers accounting for a substantial proportion of road trauma cases presenting to health facilities (6).

Personal protective equipment (PPE), including helmets, protective jackets, gloves, and boots, plays a critical role in reducing the severity of injuries sustained during motorcycle crashes (7–10). Despite the proven effectiveness of PPE in preventing fatalities and serious injuries, compliance with PPE use among commercial motorcycle riders in many African settings remains suboptimal. This has been attributed to factors such as limited awareness, negative attitudes, poor enforcement of road safety regulations, and economic constraints (11, 12).

Understanding the knowledge and attitudes of commercial motorcycle riders toward PPE is essential for designing targeted interventions that promote safe behaviors. While previous studies in Cameroon have examined the epidemiology of motorcycle-related injuries, few have focused on behavioral determinants such as knowledge and attitudes regarding PPE, particularly in high-risk districts such as Limbe and Tiko. These urban centers in the Southwest Region experience high volumes of motorcycle traffic because they are the two health districts least affected by the ongoing sociopolitical crisis in the two English-speaking regions (Northwest and Southwest) of Cameroon (13).

The Limbe and Tiko Health Districts, located in the Southwest Region of Cameroon, have been significantly affected by the ongoing sociopolitical crisis in English-speaking regions of the country (14). The crisis has disrupted socioeconomic activities, displaced populations, and strained healthcare services. In such a context, commercial motorcycle riding has become a primary livelihood strategy for many people, including internally displaced persons (IDPs) and unemployed youth. Many displaced riders in this area have little knowledge of safety measures on highways. Personal protective equipment (PPE), such as helmets, gloves, and protective clothing, is essential for mitigating these risks (15).

This study seeks to address this gap by assessing the knowledge and attitudes of commercial motorcycle riders toward the use of PPE and their associated factors in the Limbe and Tiko health districts. The findings from this study will inform evidence-based strategies to improve safety practices among riders and contribute to broader efforts in reducing motorcycle-related injuries in Cameroon.

## Materials and methods

### Study area

The study was conducted over 3 months from the 15th of May 2024 to the 17th of August 2024 in the Limbe and Tiko Health Districts of Cameroon’s Southwest Region, which is currently affected by sociopolitical unrest. As the administrative centers of their respective districts, Limbe and Tiko are known for their economic activities, including agriculture, commerce, and tourism—particularly in Limbe, which boasts attractions such as the Limbe Botanic Garden and Wildlife Centre. The road networks in these districts are crucial for transportation and commerce, with a notable presence of motorcycle riders. Given the importance of transportation and the risks associated with road traffic, researching road traffic injury prevention among motorcycle riders in Limbe and Tiko is vital for addressing public health and safety issues in these areas.

### Study design

The study adopted a community-based cross-sectional design to assess the knowledge, attitudes, and associated factors among commercial motorcycle riders regarding PPE in the crisis-affected Limbe and Tiko health districts.

### Study population

The target population for this study comprised commercial motorcycle riders aged 18 years and older who had been active in the Limbe and Tiko health districts for at least the past 6 months. These riders were specifically chosen because of their significant exposure to road traffic hazards and their potential to benefit from health education initiatives. In total, 499 commercial motorcycle riders participated in the study.

### Inclusion and exclusion criteria

The study included commercial motorcycle riders aged 18 years and above who had been operating in the Limbe or Tiko health districts for a minimum of 6 months and who provided their consent to participate. Riders who faced language barriers or health limitations that prevented their participation were excluded from the study. These selection criteria ensured that the participants were well acquainted with the local environment and could effectively engage with the study’s objectives.

### Sample size determination

The minimum sample size was estimated using the single population proportion formula: $n=Z^{2}p(1-p)/d^{2}$ . Using a 95% confidence level (*Z* = 1.96), a conservative expected prevalence of 50% (*p* = 0.50) due to limited prior estimates in this setting, and a 5% margin of error (*d* = 0.05), the minimum sample size was n=384 . To account for potential non-response and incomplete data, we increased the target sample size by 20%, resulting in a minimum required sample of 461. We ultimately enrolled 499 participants, exceeding the minimum required sample. Because clustering was not incorporated into the sample size calculation, a design effect was not applied.

### Sampling method

A multistage sampling approach was used. First, we compiled a sampling frame of major motorcycle pick-up points in each district (Limbe: 98; Tiko: 34) obtained from rider association listings and confirmed with local leaders. To ensure broad geographic coverage and representation of diverse riding contexts, we selected a high proportion of pick-up points (80%) in each district using simple random sampling. This approach was adopted to maximize coverage across neighborhoods in a setting with high mobility and fluctuating rider presence due to insecurity. At each selected pick-up point, research assistants visited at varying times of day and different days of the week. Eligible riders present at the time of the visit were approached consecutively and enrolled after consent until the site’s allocation was reached. This approach reduced systematic exclusion of riders with different work schedules; however, we acknowledge that consecutive recruitment may still introduce selection bias. The high sampling fraction (80%) was chosen to maximize geographic coverage and represent diverse riding contexts in a setting characterized by insecurity and population mobility rather than to achieve a statistically predetermined sampling fraction. Because recruitment occurred within pick-up points, responses may have been correlated within sites. We did not apply a clustering adjustment; therefore, standard errors may be underestimated. However, the large sample size, inclusion of riders from a high proportion of pick-up points across both districts, and consistency of effect estimates across key variables suggest that the main conclusions are unlikely to be substantially affected. We have added this as a study limitation.

### Data collection tools and procedures

Structured questionnaires were utilized to gather quantitative data regarding the knowledge, attitudes, and practices of motorcycle riders. To ensure the reliability and validity of the instruments, the questionnaire was pretested among 20 commercial motorcycle riders from a neighboring community to assess clarity and cultural appropriateness. Feedback informed minor revisions to improve question wording. Content validity was reviewed by three public health experts, and internal consistency reliability yielded Cronbach’s alpha values of 0.82 for knowledge and 0.79 for attitude domains. Data collection was carried out by eight trained research assistants who employed a mobile electronic data collection tool, specifically Kobo Collect. This approach facilitated efficient data entry and management. The research assistants administered the questionnaires by posing questions to the riders in English; where necessary, translations into Pidgin English were provided to ensure comprehension and accurate responses. The structured nature of the questionnaires allowed for systematic data collection, while the training of the research assistants ensured consistency in the administration of the tool, thereby enhancing the overall quality of the data collected.

### Ethical considerations

Ethical clearance was obtained from the Institutional Review Board of the Faculty of Health Sciences of the University of Buea (2024/2490-03/UB/SG/IRB/FHS). Participation was voluntary, and written informed consent was obtained from all the respondents. Confidentiality and anonymity were maintained throughout the study. Administrative authorizations were obtained from the Department of Public Health of the University of Buea, South West Regional Delegation of Public Health and the District Health Services.

### Data management and analysis

The quantitative data collected through structured questionnaires were securely entered into a Kobo collection database, which was accessible only to the principal investigator to ensure confidentiality and data integrity. All the questions were set as mandatory in the electronic questionnaire, preventing the submission of incomplete forms and thereby minimizing missing data.

Data analysis was performed via IBM SPSS Statistics version 26.0. Knowledge of personal protective equipment (PPE) was assessed via 13 items scored dichotomously: correct answers received a score of 1, and incorrect answers received a score of 0. The maximum possible score was 13. In line with the cut-off criteria commonly applied in KAP studies, a threshold of 60% of the maximum obtainable score was used to categorize participants’ knowledge. Thus, participants scoring ≥8 were classified as having “good knowledge,” whereas those scoring <8 were classified as having “poor knowledge” (16, 17).

Attitudes toward PPE were assessed via a five-point Likert scale, with scores ranging from 0 (“strongly disagree”) to 4 (“strongly agree”). The total attitude score was obtained by summing the responses across all the attitude-related items. Consistent with the knowledge assessment, a 60% threshold of the maximum possible score was used to classify attitudes: participants scoring ≥60% were categorized as having a “positive attitude,” whereas those scoring <60% were classified as having a “negative attitude” (16, 17).

Descriptive statistics were used to summarize the sociodemographic characteristics and distributions of the knowledge and attitudes scores. Categorical variables are presented as frequencies and percentages, whereas continuous variables are summarized using means and standard deviations. Education level was categorized as no formal education, primary (grades 1–6 or equivalent to elementary level), secondary (grades 7–12 or equivalent to high school), and tertiary (any post-secondary, vocational, or university training) Bivariate analyses were first performed to assess crude associations, followed by multivariable logistic regression to identify independent factors associated with good knowledge and positive attitudes. Statistical significance was set at *p* < 0.05.

Candidate variables were selected *a priori* based on literature on motorcycle safety behaviors and PPE use, and included age, education level, marital status, years of riding experience, license ownership, prior PPE training, crash history, and displacement status. We first performed bivariate analyses to explore crude associations. Variables with *p* < 0.20 in bivariate analysis and/or strong theoretical relevance were entered into multivariable logistic regression models. We retained key potential confounders (e.g., age, education, riding experience) regardless of statistical significance. Adjusted odds ratios (aORs) with 95% confidence intervals (CIs) were reported. Model fit was assessed using the Hosmer–Lemeshow goodness-of-fit test. Multicollinearity among predictors was assessed using variance inflation factors (VIFs). A VIF > 5 was considered suggestive of problematic collinearity. All VIF values were below the threshold, indicating no evidence of problematic multicollinearity.

## Results

### Sociodemographic variables of commercial motorcycle riders

With respect to the sociodemographic characteristics of the motorcycle riders (Table 1), 242 (48.5%) were within the age range of 21–30 years, and all the riders were males, while 261 (52.3%) were single. For the highest level of education, 291 (58.3%) had attended secondary school. A total of 270 (54.1%) riders’ monthly income was between 50,000 and 100,000 XAF (1 USD = 562.18XAF), and approximately half of them perceived their income as sufficient: 259 (51.9%). With respect to riding duration, the majority had been riding for 1–5 years, and 308 (61.7%) and 297 (59.5%) reported riding primarily in both urban and rural areas, respectively. The proportion of riders who reported not having a valid motorcycle license was 339 (67.9%). With respect to smoking habits, 380 (76.2%) reported that they did not smoke, whereas with respect to alcohol consumption, the majority of riders reported drinking alcohol [341 (68.3%)]. A total of 314 (62.9%) were not internally displaced. Finally, with respect to having a resting day, the majority of riders indicated that they do have a resting day, 425 (85.2%).

**Table 1:** Sociodemographic variables of motorcycle riders.

Variable	Category	Frequency	Percentage
Age group (years)	21–30	242	48.5
	31–40	192	38.5
	41–50	57	11.4
	50+	8	1.6
	Total	499	100
	Male	499	100
	Total	499	100
Marital status	Single	261	52.3
	Married	228	45.7
	Divorced	10	2.0
	Total	499	100
Highest level of education	No formal	19	3.8
	Primary	149	29.9
	Secondary	291	58.3
	Tertiary	40	8.0
	Total	499	100
Average monthly income (XAF1000) (1USD = 562.18XAF)	<50	20	4.0
	50–100	270	54.1
	101–150	158	31.7
	150+	51	10.2
	Total	499	100
Income perception	Insufficient	220	44.1
	Sufficient	259	51.9
	Largely sufficient	20	4.0
	Total	499	100
Riding duration (years)	1–5	308	61.7
	11–15	45	9.0
	15+	20	4.0
	6–10	126	25.3
	Total	499	100
Riding location	Rural	14	2.8
	Urban	188	37.7
	Both	297	59.5
	Total	499	100
Primary riding location	No	102	20.4
	Yes	397	79.6
	Total	499	100
Own a valid license	No	339	67.9
	Yes	160	32.1
	Total	499	100
Smoker	No	380	76.2
	Yes	119	23.8
	Total	499	100
Drink alcohol	No	158	31.7
	Yes	341	68.3
	Total	499	100
Internally displaced person	No	314	62.9
	Yes	185	37.1
	Total	499	100
Have a resting day	No	74	14.8
	Yes	425	85.2
	Total	499	100

*Education levels were classified as follows.

No formal education: no attendance at school.

Primary: completion of 1–6 years of schooling (equivalent to elementary education).

Secondary: completion of 7–12 years of schooling (equivalent to high school).

Tertiary: any post-secondary education, including vocational, college, or university training.

### Knowledge of commercial motorcycle riders on personal protective equipment

With respect to knowledge of commercial motorcycle riders on personal protective equipment (Table 2), a total of 380 (76.2%) knew that the primary purpose of wearing a helmet was to protect the head in the case of a road traffic crash, and 204 (40.9%) knew that the primary benefit of wearing gloves was to reduce the risk of hand injury in the case of a road traffic crash due to a fall. The majority of the riders (369, 73.9%) knew that the recommended shoes for commercial motorcycle riders were closed-toe shoes, and 388 (77.8%) knew that the primary purpose of wearing eyeglasses while riding was to protect their eyes from debris, dust or insects. A majority of the riders (320, 64.1%) correctly reported that the correct recommendation of wearing was to wear it on every ride, and 248 (49.7%) reported that their personal protective equipment should be inspected only when they notice a problem. A total of 219 (43.9%) reported that the overall primary benefit of wearing personal protective equipment was to reduce the risk of injury in cases of road traffic crashes or falls.

**Table 2:** Knowledge of commercial motorcycle riders on personal protective equipment.

Variable	Category	Frequency	Percentage
Primary purpose of wearing helmet	To attract passengers	55	11.0
	To beautify the bike	25	5.0
	To increase visibility	39	7.8
	To protect the head in case of a road traffic crash or fall	380	76.2
	Total	499	100
Primary benefit of gloves	I do not know	20	4.0
	Improve grip on the steering	89	17.8
	Make hands warm in cold weather	186	37.3
	Reduce risk of hand injury in case of a road traffic crash or fall	204	40.9
	Total	499	100
Recommended shoes for bike riders	Any shoe type is okay	95	19.0
	Closed toe shoes	369	73.9
	Sandals	33	6.6
	Slippers	2	0.4
	Total	499	100
Purpose of wearing eye wears	To attract passengers	43	8.6
	To make the rider look profession	36	7.2
	To protect the eyes from debris/dust and insects	388	77.8
	To reduce visibility	32	6.4
	Total	499	100
Recommendation for wearing PPE	Wear PPE in cold weathers to keep you warm	25	5.0
	Wear PPE on every ride, regardless of distance or location	320	64.1
	Wear PPE only during long rides	92	18.4
	Wear PPE only in urban areas	62	12.4
	Total	499	100
When to inspect PPE	Before every ride	147	29.5
	Never	12	2.4
	Once a year	92	18.4
	When they notice a problem	248	49.7
	Total	499	100
Primary benefit of wearing PPE	Attract passengers	102	20.4
	Improve comfort while riding	89	17.8
	Look responsible and professional	89	17.8
	Reduce risk of injury in case of road traffic crash	219	43.9
	Total	499	100

### Motorcycle riders’ knowledge of the different types of PPE

**Figure 1:**
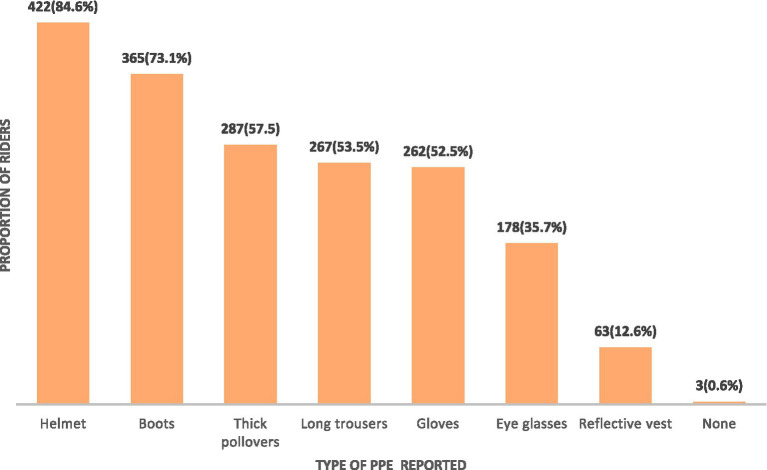
Motorcycle riders’ knowledge of the different types of personal protective equipment.

Finally, regarding the knowledge of riders of the different types of personal protective equipment, 422 (84.6%) correctly reported a helmet as a type of personal protective equipment (Figure 1).

### Overall, knowledge of bike riders on personal protective equipment

**Figure 2:**
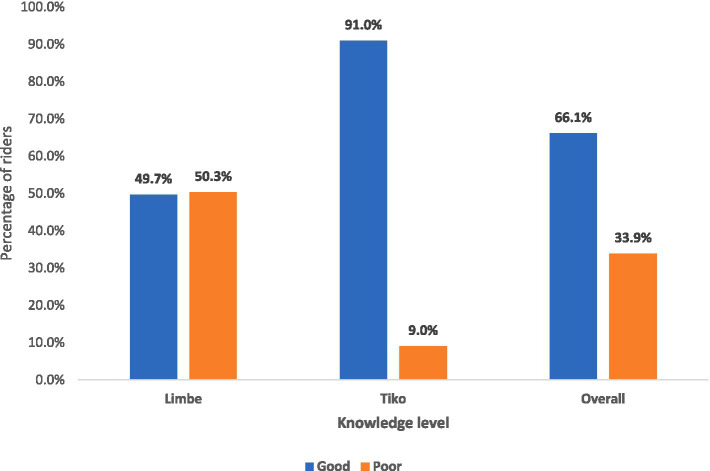
Overall knowledge of bike riders on personal protective equipment.

With respect to the overall knowledge of riders on personal protective equipment (Figure 2), the composite scoring system was used. A total of nine questions were asked with a maximum obtainable score of 13. A 60% cut-off was used to classify riders as having overall good or poor knowledge. Therefore, motorcycle riders who scored 8 or above were classified as having good knowledge, and those who scored below 8 were classified as having poor knowledge. According to this classification, the overall percentage of riders with good knowledge of personal protective equipment in the two districts was 66.1%. The Tiko Health District reported an overall good level of knowledge of 91.0%, and Limbe Health District reported a good level of knowledge of 49.7%.

### Attitudes of commercial motorcycle riders toward personal protective equipment

With respect to the attitudes of commercial motorcycle riders toward personal protective equipment (Table 3), 216 (43.3) agreed that helmets were the most important personal protective equipment for motorcycle riders, and 266 (53.3%) agreed that close-to-e-shoes were important for motorcycle riders. A total of 273 (54.7%) agreed that wearing protective eyes was important for motorcycle riders, and 257 (51.5%) agreed that wearing personal protective equipment even if not wearing it was not punishable by law. More than half of the riders (270, 54.7%) agreed that they were comfortable with wearing personal protective equipment while riding, and 295 (59.1%) agreed that wearing personal protective equipment by commercial motorcycle riders was mandatory and not a personal choice. A total of 285 (57.1%) reported feeling comfortable with the enforcement of wearing personal protective equipment by riders, and 304 (19.6%) agreed that wearing personal protective equipment while riding was generally important overall.

**Table 3:** Attitudes of commercial motorcycle riders toward personal protective equipment.

Variable	Strongly disagree	Disagree	Neutral	Agree	Strongly agree
Helmet is important to riders	5(1.0)	61(12.2)	116(23.2)	216(43.3)	101(20.2)
Closed-toe-shoes are important to riders	0(0)	37(7.4)	66(13.2)	266(53.3)	130(26.1)
Protective eye wears are important to riders	6(1.2)	44(8.8)	86(17.2)	273(54.7)	90(18.0)
Protective eye wears are important to riders	6(1.2)	44(8.8)	86(17.2)	273(54.7)	90(18.0)
Will wear PPE even if not punishable	7(1.4)	60(12.0)	110(22.0)	257(51.5)	65(13.0)
I feel comfortable when wearing PPE	2(0.4)	48(9.6)	89(17.8)	270(54.1)	90(18.0)
Wearing PPE is a necessity not a choice	5(0.8)	45(9.0)	69(13.8)	295(59.1)	85(17.0)
Feel comfortable with enforcement of PPE	4(0.8)	50(10.0)	93(18.6)	285(57.1)	67(13.4)
I think the use of PPE is generally important	2(0.4)	27(5.4)	68(13.6)	304(60.9)	98(19.6)

### Overall attitudes toward personal protective equipment

**Figure 3:**
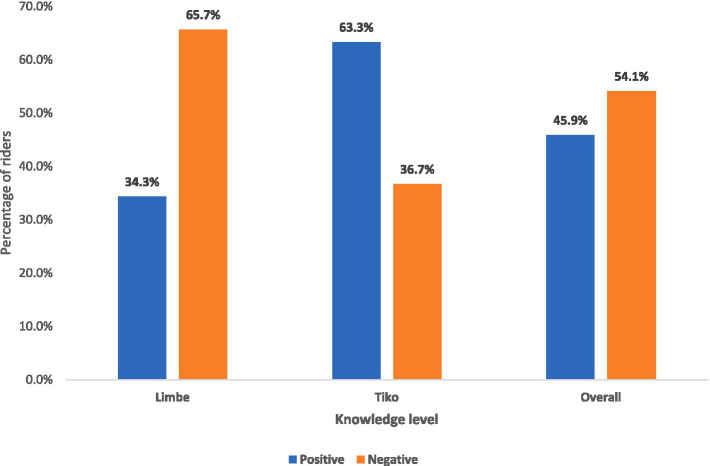
Overall attitudes of motorcycle riders toward personal protective equipment.

With respect to the overall attitudes of commercial motorcycle riders toward personal protective equipment (Figure 3), a total of nine questions were asked in this section, with a maximum obtainable score of 36. The options for the questions ranged from strongly agree (a score of 4) to strongly disagree (a score of 0). The cut-off score (60% of the maximum obtainable score) was 22. Therefore, commercial motorcycle riders who scored 22 or above were classified as having positive attitudes, and those who scored below 22 were classified as having negative attitudes. Overall, 45.9% of commercial riders had positive attitudes in both health districts. Among those in Tiko Health District, 63.3% had positive attitudes, and 34.3% had positive attitudes in Limbe Health District.

### Factors associated with knowledge of PPE among commercial motorcycle riders

With respect to factors associated with the knowledge of motorcycle riders on personal protective equipment (Table 4), three factors were found to be significantly associated with knowledge at the level of bivariable analysis. These factors included license ownership, internal displacement, and training on PPE.

**Table 4:** Factors associated with knowledge via simple logistic regression.

Variable	Category	Knowledge		COR	95% CI for COR		*p*-value
		Poor	Good		Lower	Upper	
Age group (years)	50+	2(0.4)	6(1.2)	1.9	0.5	8.3	0.374
	41–50	12(2.4)	45(9)	1.3	0.7	2.3	0.388
	31–40	73(14.6)	119(23.8)	0.9	0.6	1.3	0.457
	21–30	82(16.4)	160(32.1)	1			
Motorcycle owner	Yes	139(27.9)	258(51.7)	0.8	0.5	1.3	0.351
	No	30(6)	72(14.4)	1			
Owned a valid motorcycle license	Yes	45(9)	115(23)	2.1	1.4	3.0	**<0.001**
	No	124(24.8)	215(43.1)	1			
Are internally displaced	Yes	72(14.4)	113(22.6)	0.7	0.1	0.9	**0.040**
	No	97(19.4)	217(43.5)	1			
Trained on the importance of PPE	Yes	37(7.4)	122(24.4)	2.4	1.6	3.5	**<0.001**
	No	132(26.5)	208(41.7)	1			

Bold values indicate statistically significant associations at *p* < 0.05. OR: Odds Ratio; AOR: Adjusted Odds Ratio; CI: Confidence Interval.

License ownership, internal displacement, and training on PPE were significantly associated with factors independently associated with knowledge (Table 5). Motorcycle riders with a valid motorcycle license were more likely to have good knowledge of PPE than those with a valid license were (AOR: 1.7: 95% CI: 1.1–2.6; *p* = 0.008). Those who were internally displaced as a result of the sociopolitical crisis affecting the northwestern and southwestern regions of Cameroon were less likely to have good knowledge than those who were not internally displaced (AOR: 0.6, 95% CI: 0.4–0.9; *p* = 0.042). Finally, those who had been trained on the importance of PPE were more likely to have good knowledge than those who had not been trained (AOR: 2.0, 95% CI: 1.4–3.0; *p* = 0.001).

**Table 5:** Factors associated with knowledge via multiple logistic regression.

Variable	Category	Knowledge		AOR	95% CI for AOR		*p*-value
		Poor	Good		Lower	Upper	
Owned a valid motorcycle license	Yes	45(9)	115(23)	1.7	1.1	2.6	0.008
	No	124(24.8)	215(43.1)	1			
Are internally displaced	Yes	72(14.4)	113(22.6)	0.6	0.4	0.9	0.042
	No	97(19.4)	217(43.5)	1			
Trained on the importance of PPE	Yes	37(7.4)	122(24.4)	2.0	1.4	3.0	**0.001**
	No	132(26.5)	208(41.7)	1			

Bold values indicate statistically significant associations at *p* < 0.05. OR: Odds Ratio; AOR: Adjusted Odds Ratio; CI: Confidence Interval.

### Factors associated with attitudes of commercial motorcycle riders toward PPE use

With respect to the factors associated with the attitudes of motorcycle riders toward PPE in the bivariable analysis (Table 6), three factors were significantly associated with attitudes. These factors included motorcycle license ownership, training on PPE, and knowledge of PPE.

**Table 6:** Factors associated with attitudes via simple logistic regression.

Variable	Category	Attitudes		COR	95% CI for COR		*p*-value
		Poor	Good		Lower	Upper	
Age group (years)	50+	3(0.6)	5(1)	1.5	0.3	7.8	0.603
	41–50	27(5.4)	30(6)	1.9	1.0	3.8	0.064
	31–40	110(22)	82(16.4)	0.8	0.6	1.2	0.372
	21–30	130(26.1)	112(22.4)	1			
Marital status	Widowed	5(1)	5(1)	1.2	0.3	4.7	0.807
	Married	127(25.5)	101(20.2)	1.0	0.7	1.4	0.908
	Single	138(27.7)	123(24.6)	1			
Education	Tertiary	19(3.8)	21(4.2)	1.2	0.3	4.3	0.748
	Secondary	156(31.3)	135(27.1)	0.6	0.2	1.8	0.377
	Primary	82(16.4)	67(13.4)	0.7	0.2	2.1	0.565
	No formal	13(2.6)	6(1.2)	1			
Motorcycle owner	Yes	219(43.9)	178(35.7)	0.8	0.5	1.2	0.287
	No	51(10.2)	51(10.2)	1			
Had a valid motorcycle license	Yes	67(13.4)	93(18.6)	1.5	1.1	2.2	**0.043**
	No	203(40.7)	136(27.3)	1			
An IDP	Yes	91(18.2)	94(18.8)	0.7	0.5	1.0	0.068
	No	179(35.9)	135(27.1)	1			
Trained on PPE	Yes	63(12.6)	96(19.2)	2.1	1.4	3.2	**0.001**
	No	207(41.5)	133(26.7)	1			
Knowledge	Good	125(25.1)	205(41.1)	9.9	6.1	16.1	**<0.001**
	Poor	145(29.1)	24(4.8)	1			

Bold values indicate statistically significant associations at *p* < 0.05. OR: Odds Ratio; AOR: Adjusted Odds Ratio; CI: Confidence Interval.

At the level of multivariable analysis via multiple logistic regression (Table 7), four factors were significantly associated. These were motorcycle license ownership, internal displacement, training on PPE, and knowledge of PPE. Compared with those who did not have valid licenses, those who had valid licenses were more likely to have positive attitudes toward PPE (AOR: 1.8, 95% CI: 1.1–2.8; *p* = 0.015). Those who were internally displaced were less likely to have positive attitudes toward PPE than those who were not displaced (AOR: 0.6, 95% CI: 0.4–0.9; *p* = 0.039). Riders who had been trained on PPE were more likely to have positive attitudes toward PPE than those who had not been trained (AOR: 1.7, 95% CI: 1.1–2.6; *p* = 0.024). Those who had good knowledge of PPE were more likely to have positive attitudes than those with poor knowledge (AOR: 10.4, 95% CI: 6.3–17.3; *p* < 0.001).

**Table 7:** Factors associated with knowledge via multiple logistic regression.

Variable	Category	Attitudes		AOR	95% CI for AOR		*p*-value
		Poor	Good		Lower	Upper	
Motorcycle owner	Yes	219(43.9)	178(35.7)	0.8	0.5	1.2	0.287
	No	51(10.2)	51(10.2)	1			
Have a motorcycle license	Yes	67(13.4)	93(18.6)	1.8	1.1	2.8	**0.015**
	No	203(40.7)	136(27.3)	1			
An IDP	Yes	91(18.2)	94(18.8)	0.6	0.4	0.9	**0.011**
	No	179(35.9)	135(27.1)	1			
Trained on PPE	Yes	63(12.6)	96(19.2)	1.7	1.1	2.6	**0.024**
	No	207(41.5)	133(26.7)	1			
Knowledge	Good	125(25.1)	205(41.1)	10.4	6.3	17.3	**0.000**
	Poor	145(29.1)	24(4.8)	1			

Bold values indicate statistically significant associations at *p* < 0.05. OR: Odds Ratio; AOR: Adjusted Odds Ratio; CI: Confidence Interval.

## Discussion

This study assessed the knowledge and attitudes of commercial motorcycle riders regarding personal protective equipment (PPE) in the Limbe and Tiko Health Districts of Cameroon and examined factors associated with these outcomes. While two-thirds of riders demonstrated good knowledge, fewer than half expressed positive attitudes, highlighting a gap between awareness and perception. Several sociodemographic and contextual factors, including license ownership, PPE training, and displacement status, were found to be associated with knowledge and attitudes.

### Level of knowledge of PPE among riders and associated factors

Our study revealed that 66.1% of riders had good knowledge of PPE, which is encouraging compared with earlier findings in Cameroon, where only approximately 30% demonstrated adequate knowledge (18). This suggests potential progress in exposure to safety information, possibly due to union meetings, ongoing health education, or spillover from broader road safety campaigns.

However, one-third of riders still lack sufficient knowledge, representing a critical gap in the understanding of basic protective measures. Similar challenges have been documented in Nigeria, where less than half of riders demonstrated good knowledge of safety equipment (19). In Ethiopia, it has been reported that knowledge levels are strongly tied to exposure to road safety training (20). These comparisons highlight that despite some improvements, knowledge gaps remain a persistent barrier to safe riding practices in Sub-Saharan Africa.

Among the factors associated with knowledge of PPE, license ownership and prior training on PPE were significant predictors of good knowledge in this study. This finding is intuitive, as licensing processes typically involve exposure to traffic regulations, and structured training provides riders with information on proper safety behaviors. Similar associations between training and improved knowledge have been documented in Ethiopia (20) and Uganda (21).

On the other hand, being an internally displaced person reduces the odds of good knowledge. This reflects the broader effects of sociopolitical instability, where displacement interrupts access to formal education, licensing, and community safety networks. Similar findings have been reported in displaced populations in South Sudan and the Democratic Republic of the Congo, where conflict disrupts not only health services but also access to safety training and resources (22). This highlights the importance of tailoring safety interventions for displaced populations.

### Attitudes toward PPE among riders and associated factors among commercial motorcycle riders

Despite relatively high knowledge, only 45.9% of riders demonstrated positive attitudes toward PPE. This disconnect illustrates the well-documented gap between awareness and perception. Riders may understand the importance of PPE but perceive its use as inconvenient, uncomfortable, or financially burdensome. Attitudinal barriers such as discomfort in hot weather, the cost of helmets, and peer influence have been reported in Ghana, Benin, and Nigeria (23, 24).

The low proportion of riders with positive attitudes is concerning because attitudes often serve as a stronger predictor of behavior than does knowledge alone, as described in the knowledge, attitudes, and practices (KAP) model. This implies that interventions must not only increase awareness but also address misconceptions, affordability issues, and cultural perceptions surrounding PPE.

Several factors predicted positive attitudes in our study. Riders with a valid license and those who had received PPE training were significantly more likely to hold favorable views. This mirrors findings from Ethiopia, where training improved both knowledge and attitudes toward safety compliance (29). Riders with good knowledge were more than 10 times more likely to report positive attitudes. This strong association may partly reflect the conceptual proximity between knowledge and attitudes in safety behavior frameworks, as individuals who understand the benefits of PPE may also express favorable perceptions of its use. However, given the cross-sectional design, the direction of this relationship cannot be established, and the finding should be interpreted as an association rather than evidence of a causal pathway. However, consistent with the knowledge analysis, internal displacement reduced the odds of positive attitudes. Displaced riders may prioritize income generation over safety, or they may perceive PPE as unaffordable or inaccessible. Evidence from humanitarian contexts shows that displacement exacerbates economic vulnerability, which in turn weakens preventive behaviors (25). This finding reinforces the need for targeted support for displaced populations.

### Comparative interpretation

The level of PPE knowledge observed in this study (~66%) is higher than that reported in several comparable African contexts. For example, studies in Nigeria have found that only about one-third of commercial motorcyclists possess adequate knowledge of protective devices (19, 26). However, attitudinal barriers persist across the region, with discomfort, cost, and perceived inconvenience commonly cited as obstacles to PPE use (19). These similarities suggest that while awareness may be improving, perceptual and structural barriers continue to limit effective adoption of safety measures (26).

### Determinants and theoretical insights

License ownership and prior PPE or safety-training have been shown in other studies to correlate with better knowledge and attitudes. For example, in the Nigerian crash helmet study, formal training was associated with higher knowledge and practice (26). Internal displacement as a negative predictor is less frequently examined in road safety studies, particularly among commercial motorcycle riders in conflict-affected settings. The social instability and economic pressures associated with displacement likely disrupt access to training and enforcement, as suggested also in vulnerability literature among internally displaced populations in Africa (27). This highlights intersections between public health, social vulnerability, and occupational risk.

### Policy and regional implications

These findings add to the evidence base supporting the *Decade of Action for Road Safety 2021–2030*, which aims to reduce road traffic deaths by at least 50% (28). Integrating PPE education into licensing and formal training programs aligns with WHO recommendations and the Global Plan for the Decade of Action (28). Moreover, Cameroon’s road safety strategy would be strengthened by including specific provisions for displaced riders, subsidized PPE, union-led safety training, and better enforcement of licensing. Regional lessons from other African countries suggest that such combined policy and behavioral interventions are more effective than awareness alone.

### Strengths and limitations

This study contributes important evidence from a crisis-affected setting and includes a relatively large sample of commercial motorcycle riders drawn from multiple pick-up points across two health districts. However, several limitations should be considered. The cross-sectional design precludes causal inference, and the observed associations may reflect unmeasured confounding or reverse relationships. Data were self-reported and may be subject to recall or social desirability bias. In addition, recruitment at motorcycle pick-up points may have introduced clustering and selection bias, and the findings may not be generalizable to riders outside the study districts or to non-commercial motorcyclists.

Despite these limitations, the study provides robust and policy-relevant insights. The relatively large sample size, broad geographic coverage of recruitment sites, use of standardized data collection procedures, and multivariable analysis to control for potential confounders strengthen the reliability of the findings. Moreover, the consistency of associations across key variables suggests that the results are unlikely to be solely attributable to sampling or measurement bias. These findings therefore offer valuable context-specific evidence for informing road safety interventions in similar conflict-affected settings.

## Conclusion

The study revealed that although knowledge of PPE among commercial motorcycle riders was relatively high, attitudes remain insufficient, which may compromise actual PPE use. License ownership and training were associated with more favorable outcomes, whereas displacement consistently corresponded with poorer knowledge and attitudes. Interventions aimed at strengthening licensing, providing continuous PPE training, and targeting displaced populations may help reduce injuries and improve road safety among motorcycle riders in Cameroon. The study provides context-specific yet transferable evidence to guide road safety strategies across similar settings in Sub-Saharan Africa.

## Data Availability

The original contributions presented in the study are included in the article/Supplementary material, further inquiries can be directed to the corresponding author.

## References

[1] Chang F-R Huang H-L Schwebel DC Chan AHS Hu G-Q. Global road traffic injury statistics: challenges, mechanisms and solutions. Chin J Traumatol. (2020) 23:216–8. doi: 10.1016/j.cjtee.2020.06.001 32680705 PMC7451583

[2] World Health Organization. Global Status Report on Road Safety 2018.World Health Organization (2019). Geneva: Switzerland.

[3] Kwaghgba G Aondofa T. The effect of economic policies on urban transport patterns: a review of motorcycle usage for urban mobility in Nigeria. J Sustain Dev Transp Logist. (2024) 9:32–42. doi: 10.14254/jsdtl.2024.9-2.3

[4] Neki K Mitra S Wambulwa WM Job RFS. Profile of low and middle-income countries with increases versus decreases in road crash fatality population rates and necessity of motorcycle safety. J Saf Res. (2023) 84:129–37. doi: 10.1016/j.jsr.2022.10.014, 36868641

[5] Kemajou A Jaligot R Bosch M Chenal J. Assessing motorcycle taxi activity in Cameroon using GPS devices. J Transp Geogr. (2019) 79:102472. doi: 10.1016/j.jtrangeo.2019.102472

[6] Wankie C. Epidemiology of Crashes and Injuries among Commercial Motorcyclists in Bamenda, Cameroon: A Cross-Sectional Study. San Diego: University of California (2019).

[7] De Rome L Ivers R Fitzharris M Du W Haworth N Heritier S . Motorcycle protective clothing: protection from injury or just the weather? Accid Anal Prev. (2011) 43:1893–900. doi: 10.1016/j.aap.2011.04.027, 21819816

[8] Wu D Hours M Ndiaye A Coquillat A Martin J-L. Effectiveness of protective clothing for motorized 2-wheeler riders. Traffic Inj Prev. (2019) 20:196–203. doi: 10.1080/15389588.2018.1545090, 30901230

[9] Natarajan G Rajan TP. Review on the performance characteristics and quality standards of motorcycle protective clothing. J Ind Text. (2022) 51:7409S–27S. doi: 10.1177/15280837211014752

[10] Obst M Rzepczyk S Głowiński S Żaba C. Motorbike protective helmets, construction, testing and its influence on the type and severity of injuries of motorbike accident casualties: a literature review. Vibr Phys Syst. (2023) 34. doi: 10.2478/vps-2023-0003

[11] Umar Z Aslam R Zaka S. A review of global and local perspectives on drivers’ knowledge, attitudes, and practices towards road safety. Bull Bus Econ. (2024) 13:429–35.

[12] Heydari S Hickford A McIlroy R Turner J Bachani AM. Road safety in low-income countries: state of knowledge and future directions. Sustainability. (2019) 11:6249. doi: 10.3390/su11226249

[13] Nwati MT. The Anglophone crisis: the birth of warlords, the impact of warlords activity on the people of north west and south west region of Cameroon. Adv Appl Sociol. (2020) 10:157–85. doi: 10.4236/aasoci.2020.105010

[14] Bang HN Balgah RA. The ramification of Cameroon’s Anglophone crisis: conceptual analysis of a looming “complex disaster emergency.”. J Int Humanitarian Action. (2022) 7:6. doi: 10.1186/s41018-022-00114-1, 37519838 PMC8784587

[15] Senaji FMA Muhonja F Maathai R. Association between utilization of personal protective equipment and prevalence of road traffic injuries amongst motorcycle users in Kibera constituency, Nairobi County Kenya. Int J Curr Sci Res Rev. (2023) 6, 3799–3806. doi: 10.47191/ijcsrr/V6-i5-39

[16] Wang L Abualfoul M Oduor H Acharya P Cui M Murray A . A cross-sectional study of knowledge, attitude, and practice toward COVID-19 in solid organ transplant recipients at a transplant center in the United States. Front Public Health. (2022) 10:880774. doi: 10.3389/fpubh.2022.880774 36211649 PMC9539443

[17] Hagan D Tarkang EE Aku FY. Compliance of commercial motorcycle riders with road safety regulations in a peri-urban town of Ghana. PLoS One. (2021) 16:e0246965. doi: 10.1371/journal.pone.0246965, 33784328 PMC8009399

[18] Ukah CE Tendongfor N Hubbard A Tanue EA Oke R Bassah N . Knowledge and attitudes of commercial motorcyclists on personal protective equipment in the crisis-affected Limbe and Tiko health districts of Cameroon toward injury prevention: a formative research study for a health education intervention. [Epubh ahead of preprint]. (2025). doi: 10.1101/2025.05.06.25327113

[19] Lawal N Oche M Isah B Yakubu A Raji M. Knowledge, attitude and compliance with safety protective measures and devices among commercial motorcyclists in Sokoto metropolis, northwestern Nigeria. J Adv Med Pharm Sci. (2018) 16:1–9. doi: 10.9734/jamps/2018/39205

[20] Teshale AA Alemu ZA. Knowledge, attitude and practice of first aid and factors associated with practice among taxi drivers in Addis Ababa, Ethiopia. Ethiop J Health Dev. (2017) 31:200–7.

[21] Wegoye P Stakeholder Communication Strategies and the Performance of Road Construction Projects (2021). Kampala, Uganda: Makerere University.

[22] Bridges B Walls N. Migration, Displacement and Education. United Nation: UNESCO Publishing (2018).

[23] Hounkpe Dos Santos B Kpozehouen A Glele Ahanhanzo Y Daddah D Lagarde E Coppieters Y. Implementation of a model of awareness-raising for taxi motorcyclists in Benin in relation to helmet use: a quasi-experimental study. BMC Public Health. (2022) 22:1424. doi: 10.1186/s12889-022-13857-8, 35883078 PMC9327388

[24] Mahdavi Sharif P Najafi Pazooki S Ghodsi Z Nouri A Ghoroghchi HA Tabrizi R . Effective factors of improved helmet use in motorcyclists: a systematic review. BMC Public Health. (2023) 23:26. doi: 10.1186/s12889-022-14893-0, 36604638 PMC9814199

[25] UNHCR. Global Trends: Forced Displacement in 2022. Geneva, Switzerland: United Nations High Commissioner for Refugees (UNHCR); 2023. Available online at: https://www.unhcr.org/global-trends-report-2022

[26] Oginni FO Ugboko VI Adewole RA. Knowledge, attitude, and practice of Nigerian commercial motorcyclists in the use of crash helmet and other safety measures. Traffic Inj Prev. (2007) 8:137–41. doi: 10.1080/15389580601058472, 17497516

[27] Pham PN Johnston L Keegan K O’Mealia T Diallo DY Vinck P. Characterization of vulnerability of internally displaced persons in Burkina Faso, Mali, and Niger using respondent-driven sampling (RDS). J Refug Stud. (2023) 36:818–41. doi: 10.1093/jrs/fead044

[28] World Health Organization. (2021). Second Decade of Action for Road Safety 2021–2030. Geneva, Switzerland: World Health Organization.

[29] Kralam K Taneepanichskul N. Knowledge, Attitude and Practice towards Personal Protective Equipment Use among Steel Industry Workers in Thailand. Journal of Health Research. (2016). 30, S161–5.

